# Transcatheter lithotripsy to facilitate post-dilatation of underexpanded aortic transcatheter heart valve

**DOI:** 10.1093/eurheartj/ehac044

**Published:** 2022-02-03

**Authors:** Igor Belluschi, Nicola Buzzatti, Paolo Denti, Francesco Maisano

**Affiliations:** Heart Valve Center, IRCCS San Raffaele University Hospital, Via Olgettina 60, 20132 Milan, Italy; Heart Valve Center, IRCCS San Raffaele University Hospital, Via Olgettina 60, 20132 Milan, Italy; Heart Valve Center, IRCCS San Raffaele University Hospital, Via Olgettina 60, 20132 Milan, Italy; Heart Valve Center, IRCCS San Raffaele University Hospital, Via Olgettina 60, 20132 Milan, Italy

A 72-year-old male, with severely calcified bicuspid aortic stenosis, was treated with a transfemoral transcatheter aortic valve implantation (Evolut Pro 29 mm; Medtronic, USA) in another institution. Intraprocedurally, post-dilatation was unsuccessful to expand the prosthesis.

Three years later, the patient remained symptomatic. The imaging showed prosthetic underexpansion due to massive aortic calcification, a 13 mmHg residual gradient and moderate-to-severe perivalvular leak (*Panels A–C*).

**Figure:**
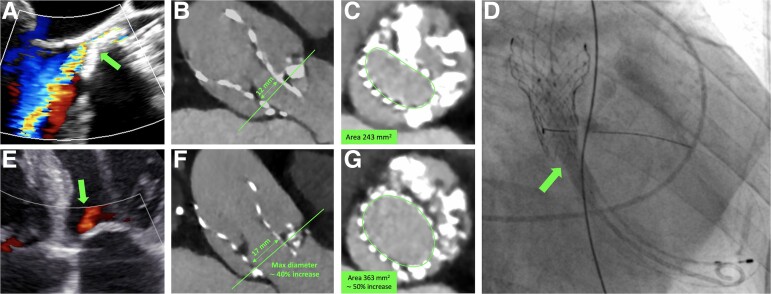


Following the Heart-team discussion, post-dilatation was planned preceded by the endovascular lithotripsy to prepare the native valve and reduce the risk of complications (annular rupture, stroke, etc.).

Under conscious sedation, the Evolut was crossed using a 5 Fr pigtail and two 0.014″ Grand Slam guides (Asahi Intecc, Japan). Two 8.0 × 60 mm Shockwave M5+ peripheral intravascular catheters (Shockwave, USA) were placed at the level of the native annulus. Under rapid pacing (160–200 b.p.m.), the balloons were simultaneously inflated at 6 atm and the pulsatile sonic waves were administered in runs of 15–30 s (*Panel D*). The patient remained stable and conscious during the rapid pacing (parallel balloons are non-occlusive). After completion of the shock cycles, the valve was successfully post-dilated with a TrueDilatation 26 × 45 mm (Bard Peripheral Vascular, USA) with a more symmetric expansion of the stent. Post-procedural gradient decreased to 6 mmHg and the paravalvular leak to mild to moderate (*Panel E*). The patient was discharged asymptomatic after the evacuation of a pre-procedural right pleural effusion. Post-procedural CT shows a more symmetric expansion of the stent and fragmentation of the calcific native leaflets (*Panels F and G*). Percutaneous aortic lithotripsy with non-dedicated devices appears feasible and potentially useful in selected patients.

The patient has signed informed consent including consent to publish the anonymized data.

